# Impact of Chromosomal Structural Rearrangements on IVF Laboratory Outcomes in PGT-SR Cycles: A Propensity Score Matching-Based Study

**DOI:** 10.3390/life15081266

**Published:** 2025-08-11

**Authors:** Daria Marzanati, Sara D’Alessandro, Davide Gentilini, Elisa Rabellotti, Laura Privitera, Sonia Faulisi, Francesca Spinella, Anil Biricik, Ettore Cotroneo, Massimo Candiani, Luca Pagliardini, Enrico Papaleo, Alessandra Alteri

**Affiliations:** 1 Department of Brain and Behavioral Sciences, University of Pavia, 27100 Pavia, Italy; 2 Reproductive Sciences Laboratory, Obstetrics and Gynecology Unit, IRCCS San Raffaele Scientific Institute, 20132 Milan, Italy; 3 Obstetrics and Gynecology Unit, IRCCS San Raffaele Scientific Institute, 20132 Milan, Italy; 4 Bioinformatics and Statistical Genomics Unit, IRCCS Istituto Auxologico Italiano, 20095 Cusano Milanino, Italy; 5 Eurofins Genoma Group, 00138 Rome, Italy; 6 School of Medicine and Surgery, Vita-Salute San Raffaele University, 20132 Milan, Italy

**Keywords:** PGT-SR, structural rearrangements, blastocyst development rate, reciprocal translocation

## Abstract

Chromosomal structural rearrangements (SR) can impair gametogenesis, increasing the risk of embryos carrying unbalanced chromosomal content (i.e., with a gain or loss of chromosomal material). In such cases, assisted reproduction technologies (ARTs) with preimplantation genetic testing for structural rearrangements (PGT-SR) is recommended to identify embryos with a normal or balanced karyotype. However, data on IVF laboratory outcomes in this context remain limited. This retrospective cohort study analyzed 548 ART cycles, comprising 129 with PGT-SR and 419 with PGT-A, conducted at a single university-affiliated center. Following propensity score matching, laboratory outcomes were compared using logistic regression. The fertilization rate was comparable between groups, but the PGT-SR group had significantly lower blastocyst development (36.7% vs. 47.1%) and top-quality blastocyst development rates (9.6% vs. 21.1%). No significant differences were found either in the blastocyst development rate on days 5, 6, 7, or in euploidy rates. In the PGT-SR cohort, the generalized linear mixed-effects model indicated no significant effect of carrier gender on the normal/balanced blastocyst rate, while the type of SR was strongly associated with it: non-reciprocal SRs yielded a higher rate of normal/balanced blastocysts (89.9%) compared to reciprocal translocations (45.7%). These findings indicate that patients undergoing PGT-SR generate fewer blastocysts available for biopsy, and that in cases involving reciprocal translocations, the proportion of normal/balanced blastocysts suitable for transfer is significantly reduced. These results underscore the importance of personalized counseling in managing expectations and supporting informed clinical decision-making.

## 1. Introduction

Chromosomal structural rearrangements (SRs) are variations in chromosome structure. They are classified as “balanced” when all genetic material is present but rearranged, or “unbalanced” when there is a gain or loss of genetic material on one or more chromosomes. Balanced SRs include Robertsonian translocations (RobT), reciprocal translocations (RecT), inversions (Inv), and insertions. Balanced SRs occur in approximately 0.25% of the general population. Nevertheless, their frequency is considerably higher in individuals facing reproductive challenges [1,2]. For instance, balanced SRs have been reported in roughly 1.1% of patients with infertility [3], in 4.5% of couples with recurrent miscarriage [4], and as many as 9.2% of couples with a history of more than three first-trimester miscarriages [2,5].

Individuals who carry balanced chromosomal rearrangements, such as balanced translocations, typically exhibit a normal phenotype. However, they face an increased risk of infertility, recurrent miscarriage, or conceiving offspring with unbalanced chromosomal content. These reproductive issues arise from the production of gametes carrying unbalanced rearrangements during meiosis I [4,6,7,8,9].

Consequently, for affected couples, undergoing assisted reproduction technology (ART) cycles with PGT-SR can be proposed to improve the likelihood of achieving a healthy pregnancy by selecting normal or balanced embryos for transfer [10].

Limited data are available on IVF laboratory outcomes in couples undergoing PGT-SR cycles. Specifically, Insogna and co-workers demonstrated that patients with SRs exhibit similar blastocyst development but have significantly fewer usable blastocysts available for transfer compared to the preimplantation genetic testing for aneuploidies (PGT-A) group [11]. In addition, conclusive evidence regarding the influence of carrier gender and type of chromosomal SR on the development of transferable blastocysts is lacking, with existing studies yielding contradictory results [12,13,14].

The primary objective of this study was to assess whether IVF laboratory outcomes, including fertilization, top-quality blastocyst development, overall blastocyst development, and euploidy rates, differ in PGT-SR cycles compared to PGT-A cycles. Additionally, this study aimed to evaluate any correlations between PGT-SR cycle outcomes and specific factors, such as the type of chromosomal rearrangement and the parental origin of the translocation. These findings are essential for providing more precise counseling to carriers of balanced translocations and for enhancing the accuracy of outcome predictions during ART treatment programs.

## 2. Materials and Methods

### 2.1. Study Design

This retrospective, single-center cohort study of PGT-SR and PGT-A cycles was performed between January 2019 and November 2023 at IRCCS San Raffaele Scientific Institute, Milan, Italy.

### 2.2. Participants

**Figure 1:**
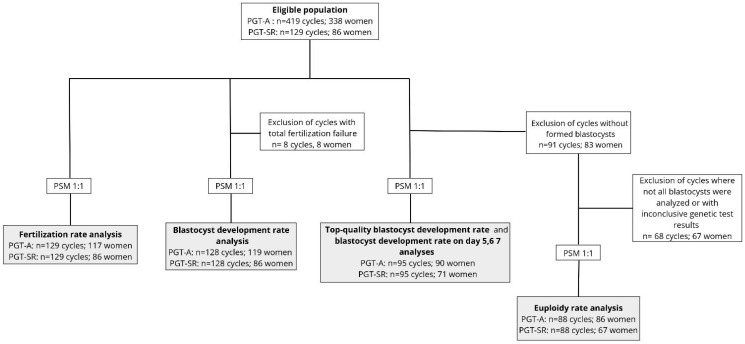
Flow chart of study design and ART cycles inclusion for the primary aim. Propensity score matching (PSM) without replacement was applied in a 1:1 ratio before assessing each IVF laboratory outcome, which are presented in the grey boxes. PGT-SR, preimplantation genetic testing for structural chromosomal rearrangements; PGT-A, preimplantation genetic testing for aneuploidies.

Female patients aged 18–42, undergoing controlled ovarian stimulation for PGT-SR or PGT-A cycles, were screened for inclusion. A detailed flow chart of the study process is shown in Figure 1. We excluded all cycles (i) with an indication for PGT for monogenic/single-gene defects (PGT-M), (ii) involving oocyte thawing, (iii) using donor gametes, and (iv) with no oocytes retrieved or degeneration of all retrieved oocytes.

Cycles in which patients were initially planning to undergo PGT-A but then opted for a fresh transfer on day 3 were excluded, to correct for the bias of transferring the best embryo on day 3, which could have developed into a blastocyst. Conversely, cycles with a fresh day-5 transfer were retained in the analysis, as they were initially scheduled as PGT-A cycles and provided unbiased evidence of blastocyst formation, thus remaining relevant for the evaluation of the primary outcome [11]. To calculate the euploidy rate, cycles were included only if PGT was actually performed, all available blastocysts were analyzed, and conclusive ploidy results were obtained for each blastocyst.

For the purposes of this study, ‘normal/balanced blastocyst’ refers to an embryo identified by PGT-SR as either chromosomally normal (without the parental SR) or balanced (carrying the same SR as the parent). These two categories were grouped together as transferable.

### 2.3. Assisted Reproductive Technology Procedures

Ovarian stimulation and trigger of ovulation strategies were chosen according to patients’ characteristics and based on gynaecologists’ judgement [15,16]. Oocyte retrieval was conducted 36 h after triggering. Intracytoplasmic sperm injection was performed as previously described by Vanni et al. [17], and embryo culture and blastocyst evaluation followed the protocol reported by Cermisoni et al. [16].

Days 5, 6, and 7 expanded blastocysts underwent trophectoderm (TE) biopsy. According to our biopsy method, a diode laser (Saturn V, Research Instruments) was used to perform artificial shrinkage on the blastocyst. After a partial expansion after collapse and when the inner cell mass (ICM) is well distinguished, a 10–20 µm hole in the zona pellucida is opened by laser, and 5–10 TE cells are aspirated by entering the zona with the pipette. After three laser pulses between the aspirated cells and the body of the embryo, the flicking method is performed in order to disaggregate the biopsy from the blastocyst mechanically. Following this, the retrieved TE fragment is expelled from the biopsy pipette to be collected. The TE fragment is washed through 10 µL drops of hypotonic solution and transferred to a PCR tube. Samples are stored at 4 °C, shipped to a reference genetic laboratory for genetic analysis (Eurofins Genoma Group, Rome, Italy).

Analysis was performed in a reference genetic laboratory (Eurofins Genoma Group, Rome, Italy), as previously published [18,19]. Immediately after biopsy, blastocysts were vitrified as described previously [16]. Following confirmation of the genetic report, biopsied blastocysts were warmed and transferred as described elsewhere [20].

### 2.4. Outcome Measures

The primary objective of this study was to compare IVF laboratory outcomes between ART cycles with PGT-A and those with PGT-SR. The IVF outcomes considered were (i) fertilization rate, (ii) blastocyst development rate, (iii) blastocyst development rate on days 5, 6, and 7, (iv) top-quality blastocyst development rate, and (v) euploidy rate.

Fertilization rate was defined as the proportion of 2PN zygotes over the total number of inseminated oocytes. The blastocyst development rate was calculated as the percentage of blastocysts formed per number of 2PN zygotes per cycle, excluding cycles with total fertilization failure. The blastocyst development rate on days 5, 6, and 7 was evaluated in cycles that yielded at least one blastocyst following fertilization and was defined as the proportion of blastocysts reaching the blastocyst stage on each day relative to the total number of blastocysts per cycle. The top-quality blastocyst rate was evaluated in cycles that yielded at least one blastocyst following fertilization and was calculated as the proportion of top-quality blastocysts out of the total number of blastocysts per cycle. Blastocyst quality was assessed based on the morphology of the trophectoderm (TE) and the inner cell mass (ICM). Blastocysts were classified as top-quality if they received one of the following scores: AA, AB, or BA. To ensure grading consistency and minimize subjectivity, blastocyst morphology was assessed by experienced embryologists. Regular internal quality control procedures, including inter-embryologist calibration sessions, were conducted within the laboratory to maintain uniformity in the evaluation of TE and ICM morphology. Finally, the euploidy rate was defined as the proportion of euploid blastocysts among the total number of blastocysts analyzed.

The second aim of this study was to evaluate, within the PGT-SR population, whether the proportion of normal or balanced blastocysts differed between cycles in which the SR carrier was male and those in which the carrier was female. A normal blastocyst was defined as having a karyotype without any SRs or chromosomal abnormalities, whereas a balanced blastocyst was defined as carrying a balanced translocation.

Only cycles that resulted in at least one blastocyst after fertilization were included. Furthermore, to reduce bias related to incomplete or inconclusive PGT results, only cycles in which all blastocysts were biopsied and yielded definitive diagnoses were included in the analysis. The rate of normal/balanced blastocysts was calculated as the proportion of blastocysts with a normal or balanced karyotype out of the total number of blastocysts analyzed per cycle.

### 2.5. Covariates

Data were obtained from medical records stored in the study center’s database. Medical history data for each couple were collected on the day of oocyte retrieval, including maternal and paternal age (years), maternal Body Mass Index (BMI; kg/m^2^), and Total Motile Sperm Count (TMSC). TMSC was categorized into three groups: ≤5 million (severe male factor), 6–19 million (moderate male factor), and ≥20 million (normal male factor). IVF cycle-related data included the length of ovarian stimulation (days), serum progesterone level on the day of ovulation trigger (ng/mL), and the proportion of mature (metaphase II) oocytes out of the total retrieved.

SRs were classified into two categories: reciprocal translocations and other types of SRs, including insertions, inversions, and Robertsonian translocations.

### 2.6. Statistical Analysis

A descriptive analysis was conducted to assess the characteristics of the study population and to evaluate differences between groups: PGT-A vs. PGT-SR, and, within the PGT-SR group, between female and male carriers of the SR.

The distribution of continuous variables was assessed using the Shapiro–Wilk test. Variables with a normal distribution within groups were described using mean and standard deviation, and comparisons were performed using the parametric *t*-test. Variables with a non-normal distribution were reported as median and interquartile range (IQR) and analyzed using the non-parametric Mann–Whitney U test. Categorical variables were presented as frequencies and percentages, and compared using the chi-square test. *p* < 0.05 was considered statistically significant.

Propensity score matching (PSM) was performed between the PGT-A and PGT-SR groups to ensure comparability of baseline characteristics. The matching was conducted using the MatchIt package in R 4.3.2 [21], applying nearest neighbor matching without replacement in a 1:1 ratio. Propensity scores were estimated through logistic regression based on couple and IVF cycle characteristics, including maternal age at oocyte retrieval, paternal age, maternal BMI, mature oocyte (metaphase II) rate, and serum progesterone concentration on the day of ovulation trigger (ng/mL). Matching quality was assessed using standardized mean differences (SMD), with an SMD < 0.2 considered indicative of acceptable comparability. Appropriate statistical tests, based on the distribution of each variable, were used to compare the two groups, and a *p* < 0.05 was considered indicative of a statistically significant difference between the groups after matching.

The effect of SRs on IVF laboratory outcomes was evaluated by comparing matched PGT-SR and PGT-A pairs using logistic regression, given the binomial distribution of the outcomes. The model was adjusted for maternal age, mature oocyte rate, serum progesterone concentration, TMSC categories, length of stimulation, and maternal BMI. To account for intra-cluster correlation introduced by the matching procedure, clustered robust standard errors were applied, using matched pair membership as the clustering variable.

For the second aim, the association between the sex of the partner carrying the SR and the rate of blastocysts with a normal or balanced karyotype was evaluated using a Generalized Linear Mixed Model with a binomial distribution, accounting for repeated measures within couples. The model was adjusted for paternal age, serum progesterone levels, the proportion of mature oocytes at the metaphase II stage, TMSC categories, type of SR, maternal BMI, and the length of ovarian stimulation.

## 3. Results

A total of 548 cycles from 424 women were included: 129 PGT-SR cycles from 86 women, and 419 PGT-A cycles from 338 women.

Before matching, significant differences were observed between the PGT-A and PGT-SR groups in maternal age, maternal BMI, and paternal age. After matching, these differences were no longer statistically significant, and the standardized mean differences (SMDs) for all three variables were below 0.2, indicating good balance between groups. The only exception was the TMSC variable in the blastocyst development rate analysis matched population, which showed a slightly higher SMD (0.201); however, the difference in the distribution of TMSC categories between the two groups was not statistically significant (*p* = 0.278) (Table S1). The results of the IVF laboratory outcome analyses comparing PGT-A and PGT-SR are reported in Table 1.

In the post-matching population, the logistic regression model revealed no statistically significant association between fertilization rate and PGT group (OR = 1.25, 95% CI: 1.00–1.55, *p* = 0.077).

**Figure 2:**
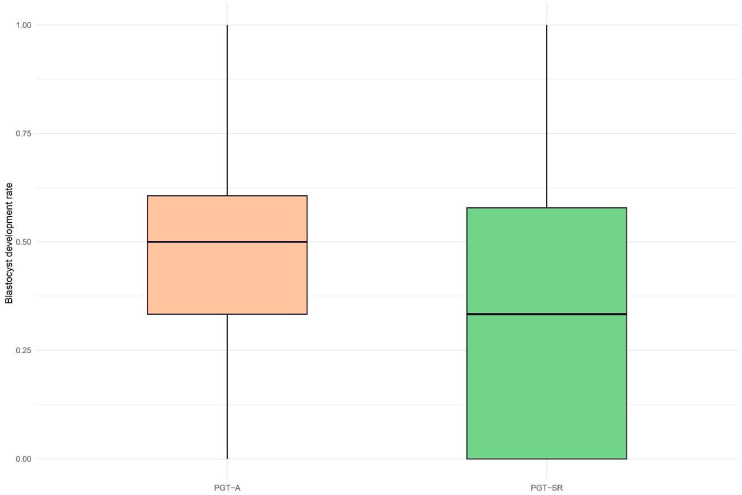
Boxplot of blastocyst development rate distribution in PGT-A and PGT-SR cycles. Blastocyst development rate was calculated as the percentage of blastocysts formed per number of 2PN zygotes per cycle.

In contrast, the blastocyst development rate was significantly lower in the PGT-SR group compared to the PGT-A group, with a median of 33.3% versus 50.0%, respectively (Figure 2). The odds of obtaining blastocysts from all fertilized zygotes were 26% lower in the PGT-SR group (OR = 0.74, 95% CI: 0.61–0.89, *p* = 0.008) compared to PGT-A. A similar trend was observed in the analysis of the top-quality blastocyst development rate with mean values of 9.6% vs. 21.1% and corresponding median values of 0.0% vs. 10%, representing a 58% reduction in the odds of obtaining top-quality blastocysts from the total number of blastocysts in the PGT-SR group (OR = 0.42, 95% CI: 0.27–0.65, *p* = 0.002). No significant associations were observed in the blastocyst development rates on days 5 (D5), 6 (D6), and 7 (D7). Specifically, the PGT-A group had a mean of 19.9% D5 blastocysts per cycle, compared with 14.2% in the PGT-SR group (OR = 0.72, 95% CI: 0.48–1.06, *p* = 0.293). For D6 blastocysts, the mean was 74.0% in the PGT-A group and 80.7% in the PGT-SR group (OR = 1.19, 95% CI: 0.83–1.70, *p* = 0.505). For D7 blastocysts, the PGT-A group had a mean of 6.1% compared with 5.1% in the PGT-SR group (OR = 1.56, 95% CI: 0.77–3.22, *p* = 0.278).

Finally, no significant association was observed in the euploidy rate, with mean values of 37.3% for PGT-SR and 37.6% for PGT-A (OR = 1.24, 95% CI: 0.88–1.75, *p* = 0.258).

The second part of this study focused exclusively on the PGT-SR population, comparing cycles in which the SR was carried by the female partner to those in which it was carried by the male partner. Descriptive analysis revealed no significant differences between the two groups in terms of maternal age, maternal BMI, length of stimulation, progesterone levels on the day of ovulation trigger, mature oocyte rate, or type of SR (Table 2).

However, a significant difference was observed in paternal age (*p* = 0.023), with a higher mean age in the male carrier group (40.2 years) compared to the female carrier group (37.3 years). Additionally, a significant difference was found in the distribution of subjects across the three TMSC categories (*p* = 0.001). In the male carrier group, the percentages were evenly distributed among the normal, moderate, and severe male factor categories (34.9%, 34.9%, and 30.2%, respectively). In contrast, the female carrier group showed a higher proportion of subjects in the normal male factor category (73.3%), with lower percentages in the moderate (15.6%) and severe (11.1%) categories.

The proportion of normal/balanced blastocysts was not significantly associated with the sex of the carrier. Conversely, a significant association was observed with the type of SR. Specifically, the presence of reciprocal translocations in the carrier’s karyotype was associated with a significantly lower likelihood of obtaining normal/balanced blastocysts (OR = 0.08, 95% CI: 0.04–0.18; *p* < 0.001), with an average rate of 45.7% among blastocysts analyzed. In contrast, cycles involving carriers of SRs other than reciprocal translocations showed a significantly higher average rate of normal/balanced blastocysts (89.9%) (Table 3).

## 4. Discussion

Our study analyzed laboratory outcomes of ART cycles by comparing couples undergoing PGT-A with couples carrying chromosomal SR who underwent PGT-SR. By applying propensity score matching, we ensured comparability between the two groups in terms of patient characteristics and IVF cycle parameters. The PGT-A population was selected as the control group to allow for comparison with a cohort of embryos that had also undergone trophectoderm biopsy, thereby eliminating the potential confounding effect of the biopsy procedure on embryological outcomes.

PGT-SR cycles showed a lower blastocyst development rate compared to the PGT-A cohort. Specifically, the average blastocyst development rate in the PGT-SR group was 36.7%, compared to 47.1% in the PGT-A group. A lower average rate of top-quality blastocyst formation was also observed in the PGT-SR group (9.6% vs. 21.1%). These differences emerged despite no significant variation between the two groups at the fertilization stage, suggesting that the divergence occurred during the subsequent phases of embryo development, particularly in the progression to the blastocyst stage.

After fertilization, the zygote undergoes mitotic cleavage, forming a morula and, subsequently, a blastocyst by approximately 116 ± 2 h post-insemination [22], a process that involves cellular differentiation and the formation of a fluid-filled cavity. According to our results, embryos derived from carriers of SR show more frequent impairment at this stage.

Several studies analyzing IVF cycle outcomes in couples carrying balanced translocations have shown that more than half of the embryos that developed to day 3 and underwent PGT-SR exhibited imbalances in the chromosomal complement [23,24]. The high rate of SRs in embryos from IVF cycles involving SR directly contributes to the reduced number of blastocysts formed. Many embryos fail to reach the blastocyst stage, likely due to natural selection mechanisms eliminating genetically non-viable embryos [25]. Beyer et al. showed that, while most embryos are unbalanced at day 3, the proportion of chromosomally normal or balanced embryos increases by day 5/6, supporting the existence of a selective process during early development [25]. Developmental arrest in early embryonic stages is often linked to reduced metabolic activity and impaired cell division. This is largely driven by the embryo’s genomic makeup, with chromosomal abnormalities such as aneuploidy, polyploidy, and mosaicism being major contributing factors [26]. When chromosomal instability becomes extensive, it ultimately leads to the failure of embryo development [27]. The elevated rate of abnormal day-3 embryos and reduced blastocyst formation in PGT-SR cycles likely reflect intrinsic genetic differences between SR carriers and PGT-A patients. Unlike PGT-A patients, SR carriers have an increased risk of generating embryos with unbalanced karyotypes, leading to chromosomal imbalances that impair normal development.

Carriers of SRs, such as reciprocal or Robertsonian translocations and inversions, have altered chromosomal structures that, when balanced, do not affect phenotype. However, during meiosis, these rearrangements disrupt proper segregation, leading to gametes with unbalanced chromosomal content, and fertilization of such gametes often results in embryos with abnormal karyotypes. As a result, PGT-SR patients may begin IVF with a biological disadvantage compared to PGT-A patients, with a lower chance of obtaining embryos with a normal or balanced chromosomal complement. The similar euploid blastocyst rates in PGT-SR and PGT-A cycles suggest a selective mechanism during early development. SRs likely lead to the early exclusion of abnormal embryos, so those reaching the blastocyst stage tend to have more stable karyotypes. This supports the hypothesis of natural selection favoring chromosomally normal embryos between fertilization and blastocyst formation [28].

Furthermore, consistent with the findings of Insogna et al. [11], our study did not provide a statistically significant difference between PGT-A and PGT-SR in terms of the day of blastocyst development. These results suggest that blastocysts in the PGT-SR group do not exhibit delayed development compared with those in the PGT-A population.

Our study then focused on the PGT-SR population to assess which variables influenced the rate of blastocysts with a normal or balanced karyotype. Specifically, the impact of the carrier’s sex was examined, with no significant differences observed between male and female carriers. However, the literature on this topic remains inconsistent. Several studies have reported contrasting results regarding the impact of carrier sex on blastocyst outcomes. While some have found no significant association [11,29], others have observed differences in the rate of transferable blastocysts [12,14,28,30]. These discrepancies highlight the need for further research involving larger sample sizes and the use of robust statistical methods to clarify the impact of carrier sex on IVF outcomes in the PGT-SR population. Although our study employed a rigorous, confounder-adjusted analytical approach, its findings are limited by the relatively small sample size, which reduces estimate precision as reflected by the wide confidence interval. Consequently, the observed non-significant association between carrier sex and the rate of normal/balanced blastocysts should be interpreted with caution, as it remains compatible with no effect as well as with a clinically meaningful effect in either direction. Notably, our analysis suggests that male carriers may have a higher probability of producing normal/balanced blastocysts, but the uncertainty reflected in this interval, spanning potential negative, null, and positive effects, prevents firm conclusions. Descriptive analysis of the PGT-SR population revealed a significant difference in paternal age between couples in which the SR carrier was female compared to those with a male carrier, with a higher average paternal age observed in the male carrier group (40.2 vs. 37.3 years). Furthermore, a significantly higher proportion of men with a normal male factor was observed in couples where the SR carrier was female (73.3% vs. 34.9%). These differences may be attributed to the established association between advancing paternal age and the deterioration of sperm parameters, including significant reductions in semen volume, concentration, motility, morphology, and overall viability [31].

Although the role of carrier sex in IVF outcomes remains controversial, more consistent evidence has emerged regarding the impact of the specific type of SR [25,30]. In line with published data, our results confirm that IVF cycles involving carriers of reciprocal translocations (RecT) are associated with a significantly lower proportion of normal or balanced blastocysts. Specifically, RecT carriers showed an average rate of 45.7% normal/balanced blastocysts among biopsied embryos, compared to a substantially higher average of 89.9% in carriers of other SRs, primarily Robertsonian translocations, as well as insertions and inversions.

This difference is likely due to the distinct meiotic segregation patterns, which influence zygote formation when gametes with unbalanced chromosomal content are fertilized.

In carriers of RecT, the rearranged chromosomes form a quadrivalent structure during meiosis. This configuration can segregate through various modes, alternate, adjacent-1, adjacent-2, 3:1, or 4:0, resulting in gametes that may be either balanced or unbalanced [32]. Robertsonian translocations follow distinct segregation patterns due to the inability of the translocated chromosomes to pair correctly during meiosis I, resulting in the formation of a trivalent structure. Segregation can occur via an alternate pattern or via adjacent segregation, which leads to zygotes with monosomy or trisomy. In some cases, all three chromosomes may segregate together, giving rise to double trisomy or double monosomy [33]. Only alternate segregation, as in RecT, can yield normal or balanced embryos [33], but reciprocal translocations are generally associated with a higher risk of generating unbalanced embryos, mainly because of the increased complexity of meiotic segregation. While both types of rearrangements rely on alternate segregation to achieve balanced outcomes, the simpler structural configuration of Robertsonian translocations (RobT) leads to a lower overall likelihood of malsegregation events [25]. The study by Ping Yuan et al. [34] further supports this observation, reporting that couples carrying RecT exhibit higher rates of unbalanced blastocysts compared not only to RobT carriers but also to those with chromosomal inversions. More recently, a large-scale study analyzing over 30,000 embryos, combining both original and previously published data, confirmed that RobT carriers have a higher frequency of chromosomally normal or balanced embryos than those carrying reciprocal translocations [28].

PGT-SR is a reliable and widely used technique that allows for the selection of embryos with a normal or balanced karyotype, thereby reducing the risk of implantation failure, miscarriage, and chromosomal disorders at birth [25,35]. By detecting and excluding embryos with unbalanced chromosomal content, PGT-SR supports high-risk couples and increases the chances of a successful pregnancy.

While the benefits of PGT-SR are well established, it remains essential that couples receive clear and up-to-date counseling regarding the realistic outcomes of IVF. Patients undergoing PGT-SR often face significant emotional, physical, and financial burdens related to fertility treatment, particularly when their history includes previous miscarriages or the loss of a child. Notably, a substantial proportion of individuals pursuing PGT-SR do not have a history of infertility and might not have considered IVF under different circumstances. Consequently, many couples pursuing PGT-SR, particularly those without prior infertility, may have unrealistic expectations regarding the likelihood of retrieving a high proportion of genetically normal embryos [36]. For this reason, it is crucial that ART specialists, including physicians, embryologists, and genetic counselors, provide clear and realistic information during counseling. Couples should be informed about the potential reduction in the blastocyst development rate, which may result in fewer blastocysts available for biopsy, and, particularly in cases of RecT, the possibility of a lower proportion of embryos with a normal or balanced karyotype. In this context, our findings help clarify expected outcomes, supporting more informed and realistic decision-making for both clinicians and patients.

In our study, the evaluation of IVF laboratory outcomes in couples undergoing PGT-SR was enabled by the comparison with a rigorously matched control group. This matching allowed for a balanced comparison and helped minimize the risk that differences between the PGT-SR and PGT-A groups were driven by imbalances in clinical characteristics. Associations within the PGT-SR group were also assessed with appropriate adjustments for potential confounders. Although the statistical analyses followed rigorous methodologies to reduce the risk of bias, the sample size was limited due to the monocentric nature of this study and the selection of ART cycles with complete and high-quality data, which may affect the generalizability of our findings. Furthermore, clinical outcomes such as pregnancy or live birth rates were not evaluated, as this study was specifically designed to focus on laboratory outcomes and their implications for counseling. Finally, different SR types (inversions, insertions, and Robertsonian translocations) were grouped together in this analysis. While this approach preserved statistical power, it may have masked potential differences in laboratory outcomes between these subtypes. Future prospective multicenter studies with larger cohorts should aim to investigate these rearrangement types separately, as they may have distinct biological behaviors and clinical implications.

## 5. Conclusions

To the best of our knowledge, this is the first study to investigate IVF laboratory outcomes by comparing PGT-SR and PGT-A through the application of propensity score matching, enabling a robust comparison of ART cycles. Our results indicate that patients carrying SRs yield fewer embryos suitable for biopsy compared to those undergoing PGT-A. Moreover, carriers of reciprocal translocations show a reduced rate of normal or balanced blastocysts. These findings highlight the importance of comprehensive counseling and managing realistic expectations for couples pursuing PGT-SR.

## Figures and Tables

**Table 1:** Results of IVF laboratory outcome analyses after propensity score matching.

Outcome	PGT-A (Ref.)	PGT-SR	OR	95%CI	*p*-Value
Fertilization rate	83.3% [66.7%, 92.3%]	85.7% [75%, 100%]	1.25	1.00–1.55	0.077
blastocyst development rate	50.0% [33.3%, 60.6%]	33.3% [0.0%, 57.9%]	0.74	0.61–0.89	0.008
Top-quality blastocyst development rate	10% [0.0%, 33.3%]	0.0% [0.0%, 0.0%]	0.42	0.27–0.65	0.002
Euploidy rate	33.3% [0.0%, 50.0%]	33.3% [0.0%, 66.7%]	1.24	0.88–1.75	0.258

A logistic regression model adjusted for maternal age (years), mature (metaphase II) oocyte rate, progesterone concentration on day of ovulation trigger (ng/mL), TMSC categories, length of stimulation (days), and maternal BMI (kg/m^2^) was used. Clustered robust standard errors were applied to account for intra-cluster correlation resulting from the matching procedure. For each group and outcome, the median [interquartile range], odds ratio (OR), 95% confidence interval (CI), and *p*-value are reported.

**Table 2:** Clinical characteristics according to SR carrier sex.

Characteristics	Female (n = 45)	Male (n = 43)	*p*-Value
Maternal age at oocytes retrieval, years; mean (sd)	35.1 (4.4)	36.1 (3.9)	0.277
Maternal BMI, kg/m^2^; mean (sd)	22.1 (2.7)	22.6 (3.3)	0.477
Paternal age, years; mean (sd)	37.3 (5.2)	40.2 (5.7)	0.023
**Seminal quality based on TMSC; n (%)**			0.001
normal male factor	33 (73.3)	15 (34.9)	
moderate male factor	7 (15.6)	15 (34.9)	
severe male factor	5 (11.1)	13 (30.2)	
Length of stimulation, days; (median [IQR])	10.0 [9.0, 11.0]	10.0 [9.0, 11.0]	0.685
Progesterone concentration on day of ovulation trigger (ng/mL); (median [IQR])	1.1 [0.7, 1.5]	0.9 [0.8, 1.3]	0.420
Mature (metaphase II) oocyte rate; (median [IQR])	0.8 [0.7, 1.0]	0.8 [0.7, 0.9]	0.624
**Structural rearrangement; n(%)**			0.698
Other structural rearrangement	16 (35.6)	18 (41.9)	
Insertion	1 (6.2)	0 (0.0)	
Inversion	6 (37.5)	6 (33.3)	
Robertsonian translocation	9 (56.3)	12 (66.7)	
Reciprocal translocation	29 (64.4)	25 (58.1)	

Differences in maternal BMI, maternal age, and paternal age between the two groups were assessed using Student’s *t*-test. Length of stimulation, number of mature (metaphase II) oocytes, and progesterone concentration on day of ovulation trigger were analyzed using the Mann–Whitney U test. Frequencies of semen quality categories and structural rearrangement types were compared using the chi-square test.n, number of cycles; BMI, body mass index; TMSC, total motile sperm count; sd, standard deviation; IQR, interquartile range.

**Table 3:** Association of SR carrier sex and other clinical variables with normal/balanced blastocysts rate.

	OR	95%CI	*p*-Value
**Carrier**			
Female	*Ref.*		
Male	1.62	0.76–3.43	0.207
**Structural rearrangement**			
Other types of SR	*Ref.*		
Reciprocal translocation	0.08	0.04–0.18	<0.001
**Maternal characteristics**			
Maternal BMI (kg/m^2^)	1.12	0.99–1.26	0.061
Mature (metaphase II) oocyte rate	2.39	0.25–22.40	0.447
Progesterone concentration on day of ovulation trigger (ng/mL)	1.08	0.72–1.62	0.720
Length of stimulation (days)	0.91	0.73–1.13	0.381
**Paternal characteristics**			
Paternal age at oocytes retrieval (years)	0.99	0.93–1.06	0.887
Seminal quality based on TMSC			
normal male factor	*Ref.*		
moderate male factor	0.99	0.47–2.10	0.989
severe male factor	0.43	0.16–1.14	0.089

Associations were evaluated using a Generalized Linear Mixed Model with a binomial distribution. The model was adjusted for paternal age, progesterone concentration on day of ovulation trigger, the proportion of mature oocytes at the metaphase II stage, TMSC categories, type of structural rearrangement, maternal BMI, and the length of ovarian stimulation. SR, Structural rearrangement; BMI, body mass index; TMSC, total motile sperm count.

## Data Availability

The data that support the findings of this study are available from the corresponding author upon reasonable request.

## Supplementary Materials

The following supporting information can be downloaded at https://www.mdpi.com/article/10.3390/life15081266/s1, Table S1. Clinical characteristics according to the PGT group. Characteristics of the PGT-A group presented before and after propensity score matching for each IVF laboratory outcome. Continuous variables were analyzed using the Mann–Whitney U test, while categorical variables were assessed using the chi-square test. N, number of cycles; IQR, interquartile range; TMC, total motile sperm count; BMI, body mass index; SMD, standardized mean difference.

## Abbreviations

The following abbreviations are used in this manuscript:

ART	Assisted Reproduction Technology
BMI	Body mass index
CI	Confidence interval
ICM	Inner cell mass
Inv	Inversions
IVF	In Vitro Fertilization
OR	Odds ratio
PGT-A	Preimplantation genetic testing for aneuploidies
PGT-M	Preimplantation genetic testing for monogenic/single-gene defects
PGT-SR	Preimplantation genetic testing for structural rearrangements
PSM	Propensity score matching
RecT	Reciprocal translocations
RobT	Robertsonian translocations
SMD	Standardized mean difference
SR	Structural rearrangement
TE	Trophectoderm
TMSC	Total Motile Sperm Count
